# The effectiveness of audiometric evaluation in drug treatment for otospongiosis

**DOI:** 10.1590/S1808-86942012000200012

**Published:** 2015-10-20

**Authors:** Andy de Oliveira Vicente, Hélio K. Yamashita, Oswaldo Laércio Mendonça Cruz, Flavia Barros Suzuki, Norma de Oliveira Penido

**Affiliations:** a PhD on ENT at the Federal University of São Paulo – Paulista Medical School (Coordinator of the ENT Residency Program at Insttuto Cema); b Associate Professor at the Imaging Department at the Federal University of São Paulo – Paulista Medical School; c Afliated Professor at the Department of ENT and Head and Neck Surgery at Unifesp-EPM; d Speech and Hearing Therapist, MSc in Speech and Hearing Therapy at the Federal University of São Paulo; e Afliated Professor at the Department of ENT and Head and Neck Surgery at Unifesp-EPM

**Keywords:** audiometry, hearing loss, otosclerosis

## Abstract

Otospongiosis is a primary osteodystrophy of the otic capsule that affects genetically predisposed individuals and leads to a progressive hearing loss.

**Aim:**

To evaluate the applicability of audiometric evaluation during drug treatment for otospongiosis.

**Materials and Methods:**

A prospective, randomized, controlled, double-blind study involving 26 patients with clinical, audiometric and CT scan image of otosclerosis. Patients eligible for the study were divided into three groups (A, B and C) and received treatment with alendronate sodium (B), sodium fluoride (C) and placebo (A) for 6 months. After this period they were submitted to new tests.

**Results:**

There were not statistically significant differences between air and bone conduction (*gap*). We also found no differences in the speech recognition threshold (SRT) and speech discrimination (IRF) between before and after treatment.

**Conclusion:**

After six months of drug treatment the audiometric evaluation kept the same hearing thresholds, suggesting stabilization of the otospongiotic lesions.

## INTRODUCTION

Otospongiosis is a primary focal osteodystrophy of the ear capsule that affects genetically predisposed individuals and introduces a metabolic disorder on the endochondral layer of the labyrinth bone characterized by resorption and disorganized new bone growth.

This disease presents no symptoms in most cases. However, under certain circumstances, it may compromise the stapediovestibular joint and lead to progressive conductive hearing loss. In some cases, the disease may be more aggressive and injure and inner ear's sensorineural structures, thus producing mixed or even sensorineural dysacusis.

Many theories have been tried to explain the injuries to the inner ear causes by otospongiosis, such as: release of toxic enzymes into the fluids of the inner ear; vascular shunts between otosclerotic sites and labyrinthine vessels; atrophy in the organ of Corti and in the stria vascularis; hyalinization of the spiral ligament; narrowing of the cochlear lumen; distortions in the basilar membrane; and degeneration of cochlear nerve cells^1^.

The enzyme theory seems to be more widely accepted in the literature. Proteolytic and hydrolytic enzymes are released by lysosomes rupturing inside histiocytes found in the active otospongiosis site, thus adversely affecting the labyrinthine neuroepithelium^2^, ^3^, ^4^, ^5^.

Given the progressively degenerative characteristics of otospongiosis, it became necessary to resort to drugs to limit the disease's activity. The use of bone metabolism inhibitors aims at providing for the preservation of hearing thresholds (sensorineural component) and improving symptoms such as tinnitus and vertigo.

Along the years, experimental and clinical trials have tried to show the positive effects of fluoride in stabilizing hearing thresholds in patients with otospongiosis and improving associated symptoms such as tinnitus and vertigo^2^, ^5^, ^6^, ^7^, ^8^, ^9^, ^10^. Drug therapy for otospongiosis with sodium fluoride is, undoubtedly, the most widely prescribed treatment for this condition by ENT physicians. This drug principally reduces enzyme activity derived from otospongiosis, aside from diminishing the rate of osteoblast-induced bone remodelling^10^.

Shambaugh & Scott^11^ were the first to use sodium fluoride to treat osteodystrophy and propose the prescription of moderate doses of this drug to promote recalcification and deactivation of the active otospongiosis sites.

Double-blind controlled clinical trials have also shown the efficacy of sodium fluoride in stabilizing hearing thresholds (sensorineural component of hearing)^9^, ^10^, ^11^, ^12^, ^14^.

Some authors have observed that the treatment with sodium fluoride could diminish the progression of sensorineural hearing loss, mainly in higher frequencies^5^.

Given their better anti-resorption properties and closer affinity with bone tissue, biphosphonates have replaced sodium fluoride in the treatment of osteodystrophies such as Paget's disease, osteogenesis imperfecta, and osteoporosis. Following this therapeutic line, biphosphonates also began to be used to treat otospongiosis. Stutzmann & Petrovic^13^ were the first to propose that otospongiosis be treated with biphosphonates.

Biphosphonates interact with osteoclast metabolism to induce osteoclast apoptosis, thus inhibiting bone resorption. By that same mechanism, the production of toxic enzymes is reduced secondarily to abnormal bone metabolism^15^. These drugs have been considered to be the most promising medication to treat otospongiosis^16^.

Kennedy et al.^17^ carried out a double-blind prospective study to assess disodium etidronate in treating progressive hearing loss in otospongiosis patients. The group given etidronate tended to have their air conduction hearing thresholds stabilized at 1000 and 4000 Hz while bone conduction thresholds were stabilized at 500, 1000 and 2000 Hz (there was no statistically significant difference between the case and control groups).

A retrospective study done by Brookler & Tanyeri^18^ observed that etidronate (a first generation biphosphonate) appeared to be more effective in managing otoneurologic symptoms introduced by otospongiosis (hearing loss, tinnitus, and vertigo) than sodium fluoride.

An experimental trial carried out with genetically modified mice with inhibited expression of osteoprotegerin (OPG – a glycoprotein that blocks the formation of osteoclasts and inhibits osteolysis) showed that biphosphonates (zolendronate and risedronate) significantly suppressed remodelling and resorption in the ear capsule and reduced hearing loss according to hearing electrophysiological test results. The authors suggested that these drugs could be useful in preventing hearing loss associated with otosclerosis^19^.

This study aims to verify the application of audiometric assessment along with the drug therapy given to otospongiosis patients.

## MATERIALS AND METHODS

This is a randomized double-blind controlled prospective trial with 26 patients diagnosed with otospongiosis through medical, audiometric, and CT imaging examination. This study was approved by the Research Ethics Committee of our institution (CEP 0114/05), as well as the terms of the informed consent form.

The patients were submitted to medical examination (interview, ENT examination) and audiometry testing (pure-tone audiometry, speech audiometry, and impedance tests).

Patients of both genders with ages ranging between 20 and 70 years were enrolled in the study. All were medically examined and suspected for otospongiosis, had normal otoscopy findings, audiometry indicative of otospongiosis and high-resolution CT scans showing active otospongiosis. None of the patients had history of surgery in the examined ear.

Pregnancy, presence of chronic infection/inflammation, esophageal or gastric diseases, metal prosthesis, pacemakers, intolerance or adverse reactions to the prescribed medication, and use of anti-resorption or anti-enzymatic drugs 12 months prior to the study were used as exclusion criteria.

Eligible patients were randomized into three groups (A, B, and C) and were treated for six months with sodium alendronate, sodium fluoride, or placebo. Patients, treatments, and analysis were masked.

Both medication (10mg sodium alendronate and 20mg sodium fluoride) and placebo pills were produced at the Pharmaceutical Laboratory of the University of São Paulo (USP) and analyzed at the Medication, Cosmetics, and Raw Material Control Laboratory at USP (CONFAR) (Analysis Certificate nr. 1281/07 and 1674/07).

The patients underwent audiometry testing after the completion of the drug therapy.

### Audiometry Testing

Hearing thresholds by air (AC) and bone conduction (BC) before and after treatment were acquired for frequencies between 500 and 4000 Hz, and the air-bone gap was compared to the speech recognition threshold (SRT) and speech discrimination (PB max). All tests were carried out by the same speech and hearing therapist using the same equipment (Maico model MA-41).

### Statistical Analysis

Observed variables:

• Audiometry testing: hearing thresholds by air and bone conduction (in dB) were assessed before and after drug therapy, as well as air-bone gaps at four frequencies (500 Hz, 1 KHz, 2 KHz, and 4KHz), together with speech recognition thresholds (SRT) and speech discrimination (PB max).
• Statistical analysis aimed to compare the progress seen in the three study groups before and after treatment in terms of their audiometry tests.

### Statistical Assessment

The groups were compared for the observed variables at one time through Fisher's exact test for qualitative variables and by ANOVA or the Kruskal-Wallis test for quantitative variables.

Generalized estimating equation models were used to analyze pre and post-treatment quantitative variables.

Statistical significance was attributed to results whose descriptive levels (p values) were lower than 0.05.

Statistical assessment was performed with the aid of SPSS release 17.0.

## RESULTS

The study enrolled 26 patients diagnosed with otospongiosis through medical, audiometric, and CT imaging examination. Ten of these patients had undergone stapedectomy (10 ears) and these ears were excluded from the study. Forty-two ears were thus analyzed and divided into groups as follows:

### Groups

Group A: nine patients taking placebo – 1 pill/day
Group B: eight patients taking sodium alendronate – 1 pill 10 mg/day
Group C: nine patients taking sodium fluoride – 1 pill 20 mg/day

### Audiometry Testing

Hearing thresholds by air and bone conduction at 500 Hz, 1000 Hz, 2000 Hz, and 4000 Hz were acquired. Pre and post-treatment air-bone gaps, speech recognition thresholds (SRT), and speech discrimination (PB max) were analyzed.

In bone conduction, differences were observed only at higher frequencies (2000 and 4000 Hz). At 2000 Hz, Group A had a greater mean threshold after treatment than Group C (*p*=0.018). However, we must point out that such difference already existed prior to treatment (*p*=0.051). At 4000 Hz, the mean values observed in Group A were greater than those seen in Groups B and C, both pre and post-treatment. At this frequency range, only Group A presented a statistically significant difference between pre and post-treatment values (*p*=0.008) (Table 1).

**Table 1:** Hearing threshold by bone conduction (BC) – mean values and standard error (se) adjusted for the dependency between measurements in individual patients.

			Group	
	Time	A	B	C
		(n=9)	(n=8)	(n=9)
BC 500 Hz- mean (se)	Pre	31.33 (4.13)	28.18 (3.96)	30.0 (3.02)
	Post	30.33 (3.84)	27.73 (3.40)	29.33 (3.54)

BC 1K – mean (se)	Pre	34.67 (4.25)	30.91 (5.03)	29.33 (4.63)
	Post	35.00 (3.77)	30.00 (3.46)	28.67 (4.34)

BC 2K – mean (se)	Pre	44.33 (3.61)	37.27 (4.15)	34.00 (3.89)
	Post	45.00§ (2.67)	37.27 (3.44)	33.33§ (4.14)

BC 4K – mean (se)	Pre	50.00§▴* (3.06)	32.73 ▴ (6.14)	34.33§ (5.59)
	Post	46.00 ▴* (2.33)	32.27 ▴ (5.19)	35.00 (5.66)

§ *p*<0.05 when comparing A to C.

▴ *p*<0.05 when comparing A to B.

* *p*<0.05 when comparing Pre to Post.

In air conduction at 500 Hz, only Group A showed a significant difference between pre and post-treatment values (*p*<0.001). Other differences were seen at 4000 Hz, as mean hearing thresholds from Group A patients were greater than those seen on Group B before and after treatment (*p*=0.014 and *p*=0.043 respectively). A difference was also seen on Group C, albeit not statistically significant (*p*=0.083 before and *p*=0.068 after treatment). At 4000 Hz differences were seen between pre and post-treatment values only on Group C (*p*<0.001) (Table 2).

**Table 2:** Hearing thresholds by air conduction (AC) – mean values and standard error (se) adjusted for the dependency between measurements in individual patients.

			Group	
	Time	A	B	C
		(n=9)	(n=8)	(n=9)
AC 500 Hz – mean (se)	Pre	62.33* (4.23)	57.73 (3.88)	56.33 (3.82)
	Post	60.00* (3.86)	55.45 (4.76)	55.00 (4.14)

AC 1K – mean (se)	Pre	61.33 (5.26)	57.27 (4.42)	55.33 (4.79)
	Post	60.33 (4.93)	57.27 (4.99)	57.67 (68.03)

AC 2K – mean (se)	Pre	57.00 (4.66)	51.82 (4.19)	48.00 (5.60)
	Post	56.00 (3.84)	50.91 (4.46)	45.67 (5.37)

AC 4K – mean (se)	Pre	72.67 ▴ (3.92)	53.64▴ (6.71)	57.67* (7.73)
	Post	70.33▴ (3.75)	55.45▴ (6.33)	54.00* (8.14)

^§^*p*<0.05 when comparing A to C.

▴ *p*<0.05 when comparing A to B.

* *p*<0.05 when comparing Pre to Post.

Air-bone gap values did not elicit statistically significant differences (Table 3). Likewise, speech recognition thresholds (SRT) and speech discrimination (PB max) values did not present statistically significant differences.

**Table 3:** Air-bone *gap* – mean values and standard error (se) adjusted for the dependency between measurements in individual patients.

			Group	
	Time	A	B	C
		(n=9)	(n=8)	(n=9)
GAP 500 Hz – mean (se)	Pre	31.00 (3.39)	29.55 (3.19)	26.33 (2.29)
	Post	29.67 (3.57)	27.73 (3.40)	26.00 (2.31)

GAP 1K – mean (se)	Pre	26.67 (3.62)	26.36 (2.21)	26.00 (2.68)
	Post	25.33 (4.01)	27.27 (2.44)	26.67 (2.80)

GAP 2K – mean (se)	Pre	12.67 (3.41)	14.55 (2.03)	14.00 (2.08)
	Post	10.33 (3.18)	15.00 (1.58)	12.33 (2.05)

GAP 4K – mean (se)	Pre	23.00 (3.08)	20.91 (4.03)	22.67 (3.39)
	Post	24.33 (2.78)	23.18 (3.66)	20.00 (4.08)

## DISCUSSION

The ear capsule presents very little bone remodelling, mainly in the sites close to the cochlear and vestibular perilymphatic spaces (noble sensorineural areas)^20^.

Otospongiosis is a progressive chronic inflammatory disease that disrupts the labyrinth bone metabolism, possibly leading to relevant structural and functional damage to the ear capsule and the cochlear/vestibular neuroepithelium.

Predicting individual progress in this osteodystrophy can be a tall order, despite the various technological resources available today. It is estimated that 9% of the patients with otospongiosis will evolve to deep sensorineural hearing loss^1^, ^21^.

Indication of drug therapy is done on an each case basis. Otospongiosis patients presenting exacerbated signs of disease – Schwartz sign (increased vascularity of the promontory secondary to vascular congestion caused by an active otosclerotic site); onset or worsening of tinnitus and/or vertigo; and progressive worsening of sensorineural hearing component – must be promptly placed in drug therapy.

The 26 patients in the trial were randomized into three groups. Patients in Group A took placebo, those in Group B took 10mg of sodium alendronate, and Group C patients took 20mg of sodium fluoride for six months. Pills were administered in the morning with patients fasting (30 minutes before their first morning meal) to improve alendronate absorption. As it is a double-blind trial, pill administration time was standardized for everyone. Only two patients in Group C had mild epigastralgia, and were treated with 150mg of ranitidine at night. As this adverse reaction is expected, sodium fluoride was administered at 20mg/day, once higher dosages could lead to the study's early termination.

Drug therapy efficacy in treating otospongiosis patients is still controversial as to the type of medication, dosage, and duration of treatment.

Very few randomized controlled trials have looked into the efficacy of such drug therapies^9^, ^14^, ^17^ and the literature lacks studies comparing and determining the best course of therapy.

The first drug prescribed to treat otospongiosis was sodium fluoride, given its track record with other osteodystrophies such as osteoporosis and Paget's disease^16^.

According to the literature, the fluoride dosage to treat otospongiosis ranges between 20 and 40mg per day, while treatment lasts from six months to two years. This dosage is deemed safe in relation to the possible undesired effects the drug may cause, principally flu-orosis, the most severe complication in this therapy. Vitamin D and calcium supplementation to prevent bone malformation is not required in these cases^10^.

Sodium fluoride must be administered, preferably, after a copious meal (lunch or dinner) so that gastric discomfort – the most common adverse effect with this drug – is minimized. This symptom can be improved with the use of pills with enteric coating or with the concomitant administration of calcium carbonate. However, fluoride absorption is diminished in these cases^10^. Given the extremely different dosages of the two drugs used in this trial, this preventive guideline could not be enforced.

Anti-enzyme dosages of sodium fluoride (3–15mg per day) – lower than the usual therapy regimes – may be indicated to patients presenting persisting signs of disease. They may also be used safely for a number of years as maintenance therapy^10^.

Biphosphonates are slowly replacing sodium fluoride in the treatment of otospongiosis. Their more recent variations (alendronate, risedronate, and ibandronate) are more potent anti-resorption agents and do not interfere relevantly in bone mineralization when compared to etidronate. These drugs may be administered orally with the same efficiency either daily (10mg alendronate or 5mg risedronate) or once a week^15^, ^22^, ^23^.

Biphosphonate absorption is harmed when they are taken concomitantly to food, and this is why they should be preferably administered 30 minutes before the first morning meal^15^. This is why we chose to administer drugs while patients were fasting.

Biphosphonates are usually well tolerated when administered orally. The main adverse effects are nau sea, vomiting, abdominal pain, dyspepsia, diarrhea, arthralgia, and headache. Esophagitis is the most common complication, but other gastrointestinal diseases such as gastritis, gastric ulcer, and duodenitis may occur^15^.

The noteworthy scarcity of papers in the literature looking at the use of biphosphonates to treat otospongiosis makes it harder for one to structure proper, reliable therapeutic parameters. In our practice, biphosphonates are used from 6 to 12 months. The treatment can be repeated if the disease shows relevant signs of activity.

Audiometry test findings indicated a general trend towards stabilization of hearing thresholds by air and bone conduction. Hearing thresholds by bone conduction were statistically reduced at 4000 Hz among Group A patients, as were the thresholds by air conduction at 4000 Hz for Group C subjects (Tables 1 and 2). These were punctual findings probably lacking clinical relevance. Most patients also presented sensorineural involvement, clearly indicating higher disease activity. In this specific case, the maintenance of hearing thresholds during the six months of treatment points to stabilization of the injuries caused by otospongiosis. Longer treatment would certainly be needed to observe substantial improvement or more evident stabilization as seen in the audiometry tests.

There was no statistically significant difference between air-bone gap, speech recognition threshold (SRT), and speech discrimination (PB max) values when pre and post-treatment results were compared (Tables 3 and 4).

**Table 4:** Speech recognition threshold (SRT) and speech discrimination (PB max) – mean values and standard error (se) adjusted for the dependency between measurements in individual patients.

			Group	
	Time	A	B	C
		(n=9)	(n=8)	(n=9)
SRT – mean (se)	Pre	63.33 (4.23)	59.55 (4.54)	56.00 (4.41)
	Post	62.33 (3.72)	58.64 (4.67)	56.67 (4.52)

PB max – mean (se)	Pre	95.20 (0.81)	97.09 (1.09)	95.20 (1.56)
	Post	96.27 (0.88)	97.45 (1.06)	95.47 (1.69)

We must stress that treatment duration (six months) may not have been sufficient to elicit more significant improvements or stabilization of otospongiosis in the audiometry tests.

## CONCLUSION

After six months of drug therapy, hearing thresholds were maintained and hearing loss levels kept stable, probably due to a reduction in disease activity on the sites affected by otospongiosis.
